# Systematic Modification of the Glass Transition Temperature of Ion-Pair Comonomer Based Polyelectrolytes and Ionomers by Copolymerization with a Chemically Similar Cationic Monomer

**DOI:** 10.3390/gels7020045

**Published:** 2021-04-13

**Authors:** Guodong Deng, Timothy D. Schoch, Kevin A. Cavicchi

**Affiliations:** 1 School of Polymer Science and Polymer Engineering, The University of Akron, Akron, OH 44325, USA; gd28@zips.uakron.edu (G.D.); tschoch@okstate.edu (T.D.S.); 2 Promerus LLC., 225 W Bartges St, Akron, OH 44307, USA; 3 Department of Chemistry, Oklahoma State University, Stillwater, OK 74078, USA

**Keywords:** ion-pair comonomer, polyelectrolyte, ionomer, polyampholyte, glass transition temperature

## Abstract

Ion-pair comonomers (IPCs) where both the anion and cation contain polymerizable functional groups offer a route to prepare polyampholyte, ion-containing polymers. Polymerizing vinyl functional groups by free-radical polymerization produces bridging ion-pairs that act as non-covalent crosslinks between backbone segments. In particular the homopolymerization of the IPC vinyl benzyl tri*-n-*octylphosphonium styrene sulfonate produces a stiff, glassy polymer with a glass transition temperature (*T_g_*) of 191 °C, while copolymerization with a non-ionic acrylate produces microphase separates ionomers with ion-rich and ion-poor domains. This work investigates the tuning of the *T_g_* of the polyelectrolyte or ion-rich domains of the ionomers by copolymerizing with vinyl benzyl tri*-n-*octylphosphonium *p-*toluene sulfonic acid. This chemically similar repeat unit with pendant rather than bridging ion-pairs lowers the *T_g_* compared to the polyelectrolyte or ionomer containing only the IPC segments. Rheological measurements were used to characterize the thermomechanical behavior and *T_g_* of different copolymers. The *T_g_* variation in the polyelectrolyte vs. weight fraction IPC could be fit with either the Gordon–Taylor or Couchman–Karasz equation. Copolymerization of IPC with a chemically similar cationic monomer offers a viable route to systematically vary the *T_g_* of the resulting polymers useful for tailoring the material properties in applications such as elastomers or shape memory polymers.

## 1. Introduction

Ion-containing polymers have long attracted attention due to the physical properties associated with the ions (e.g., conductivity [1], glass transition [2], and dynamic bonding [3]) and their interactions with other chemical species (i.e., solvent [4], salts [5], and non-ionic repeat units [6]).

Of particular interest is the ready ability to manipulate the glass transition temperature (*T_g_*) of ion-containing polymers by manipulating the strength and connectivity of the ionic interactions. Ionic groups offer the flexibility to tune *T_g_* through mild reaction and processing conditions, such as choice of the initial ions (e.g., ammonium vs. phosphonium [7], zwitterion formation [8]), ion-exchange [9,10,11], polyelectrolyte complexation [12,13], and plasticizing with salt and water [5,14,15,16,17]. These plasticizers are especially attractive as benign additives to temper or aid in the processing of polyelectrolyte complexes [18,19].

There has been recent interest in understanding the structure-property relationships controlling *T_g_* in polyelectrolyte complexes [2,16,17,20,21,22] Oppositely charged polyelectrolytes, when mixed, form both intrinsic (i.e., chain bridging) and extrinsic (i.e., pendant) ion-pairs whose concentration may be manipulated by the concentration of water and added salt. It has recently been shown that the *T_g_* is related to the ratio of water molecules surrounding an intrinsic ion pair (*n_H2O_/n_intrinsic ion pairs_*) as *1/T_g_ ~ ln*(*n_H2O_/n_intrinsic ion pairs_*). Therefore, increasing the amount of water or decreasing the concentration of intrinsic ion pairs tends to lower the *T_g_* of the polyelectrolyte complex.

An interesting route to generate ion-containing polymers with a structure similar to a polyelectrolyte complex is through the use of ion-pair comonomers (IPCs), where both the anion and cation are attached to a polymerizable unit [23]. This produces polyampholyte chains by the statistical incorporation of the cationic and anionic units along the chain backbones and produces ion-pair crosslinks bridging two backbone repeat units (i.e., intrinsic ion pairs).

IPCs and the associated polymers were first studied in depth by Salamone and co-workers in the 1970s and 1980s [24,25,26,27,28,29]. Recently there has been renewed interest in IPCs for the synthesis of tough elastomers and hydrogels due to the dissipation of energy by the breaking and reformation of ionic bonds under load [30,31,32].

In a previous work we synthesized the IPC vinyl benzyl tri-*n*-octylphosphonium styrene sulfonate and prepared polyelectrolytes, by homopolymerization of the IPC, and *n*-butyl acrylate (BA) ionomers, by copolymerization of the IPC and BA, through reversible addition fragmentation chain transfer (RAFT) polymerization [33]. This particular IPC formed strong ion-pairs, which act similar to covalent crosslinks limiting the chain mobility and produced a high *T_g_* polymer (191 °C) [34]. A control sample of poly(vinylbenzyl tri-*n*-octylphosphonium *p*-toluene sulfonate) (PVBTOP-TS) polymerized from a monomer that is chemically identical to IPC, except for the polymerizable double bond on the anion, exhibited a much lower *T_g_* of 66 °C. This was attributed to the weaker interaction between the pendant ion-pairs (i.e., dipole–dipole interactions) in the cationic polyelectrolyte that did not have as dramatic an effect on the chain mobility as the chain-bridging ion-pairs in the polyampholyte.

**Figure 1:**
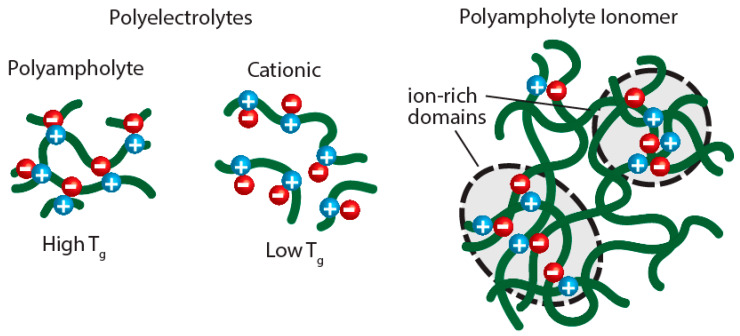
Schematic microstructure of polyelectrolytes, formed by the polymerization of an ion-pair comonomer (IPC) (polyampholyte) or a cationic monomer (cationic), and a polyampholyte ionomer formed by the polymerization of an IPC and neutral monomer.

When the IPC was copolymerized with *n*-butyl acrylate to form ionomers, microphase separation into ion-rich and ion-poor domains took place. In the ion-rich domains the *n*-butyl acrylate segments acted as a plasticizer. However, as the neat polymerized IPC had a high *T_g_*, the ion-rich domains were still glassy at room temperature even when significantly plasticized. These results are schematically summarized in Figure 1. It should be noted that while the basic driving force for ionomer self-assembly is the clustering of the ionic groups [35,36], the details of the morphological behavior and driving forces are still an active area of study [37]. For example, in these systems with more oleophilic ions the relative affinity of the non-ionic units for the different ions may drive variations in the morphology [38]. Therefore, being able to systematically tune the structure of ionomer may provide model systems for more detailed self-assembly studies.

Building on this initial work in a separate study, the same IPC was copolymerized with isobornyl acrylate (IBoA), 2-ethylhexyl acrylate (EHA), and poly(ethylene glycol) dimethacrylate by UV photopolymerization to produce crosslinked polymers [39]. During photopolymerization microphase separation occurred producing ion-rich and ion-poor domains. By varying the ratios of IBoA and EHA with 30wt% IPC it was possible to increase the *T_g_s* of both the ion-rich and ion-poor domains above room temperature to produce glassy materials with two well-separated *T_g_s.* Crosslinked, glassy polymers are known to exhibit shape memory where they can be elastically deformed above *T_g_*, fixed in the deformed shape by vitrifying under load, and then recovered by heating the unloaded sample back above *T_g_* [40]. The IBOA-EHA-IPC copolymers with two *T_g_s* are therefore able to exhibit triple shape memory, where the structure can be deformed at two different temperatures to programs a sequence of two shape transitions back to the third, original shape. This polymer formulation was then used as a resin for digital light processing (DLP) 3D printing to produce complex shapes that exhibit triple shape memory and sequential shape shifting.

A key feature of this second work was the ability to manipulate the *T_g_* of the ion-rich and ion-poor domains through the acrylate feed ratio in the copolymer. However, given the 125 °C variation in the *T_g_* of the polyelectrolytes with pendant (PVBTOA-TS) or bridged (PVBTOA-SS) ion-pairs, copolymerizing the IPC with the cationic monomer VBTOP-TS offers another route to tune the *T_g_* of the polyelectrolyte, similar to the variation in the concentration of intrinsic and extrinsic ion-pairs in polyelectrolyte complexes. While polyelectrolyte complexation provides the flexibility to tune the *T_g_* through the use of additives, such as water and salt, it is also susceptible to humidity fluctuations, which are not always an easy environmental condition to control. In contrast, direct copolymerization of an IPC and cationic monomer allows the fraction of bridging and pendant ion-pairs to be fixed in the formulation and the materials are less susceptible to humidity variations when more hydrophobic ions are used. As will be shown, this copolymerization strategy is highly effective for tuning the *T_g_* in both the polyelectrolyte and the ion-rich domains of the ionomers where the *T_g_* monotonically varies with the relative fractions of IPC and cationic monomer. Given the ease of synthesis of the IPC and its copolymerization with other monomers this formulation approach should be useful in the design of other advanced polymers including shape memory polymers, as previously mentioned, and other applications, such as high service temperature thermoplastic elastomers [41,42], where the *T_g_* and processability may be tuned through the monomer formulation.

## 2. Results and Discussion

### 2.1. Monomer and Polymer Synthesis

**Scheme 1:**
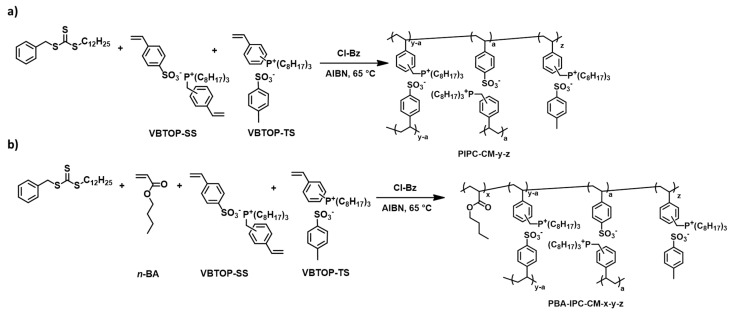
Synthetic route for (**a**) polyelectrolyte copolymers (PIPC-CM-*y*-*z*) and (**b**) poly(*n*-butyl acrylate) ionomer networks (PBA-IPC-CM-*x*-*y*-*z*)bearing different mole ratio of ionic liquid monomers: vinylbenzyl tri-*n*-octyl phosphonium 4-styrenesulfonate (VBTOP-SS) and vinylbenzyl tri-*n*-octylphosphonium *p*-toluene sulfonate (VBTOP-TS).

The ion-pair comonomer (IPC), vinylbenzyl tri-*n*-octyl phosphonium 4-styrenesulfonate (VBTOP-SS), and a chemically similar cationic monomer (CM) with a pendant ion-pair, vinylbenzyl tri-*n*-octylphosphonium *p*-toluene sulfonate (VBTOP-TS), were prepared by a two-step synthetic route as previously reported [33]. Scheme 1a depicts the synthetic routes for the preparation of polyelectrolytes through the copolymerization of the ion-pair comonomer VBTOP-SS and cationic monomer VBTOP-TS with different feed ratios using reversible addition-fragmentation chain transfer (RAFT) polymerization. As an example, VBTOP-SS and VBTOP-TS were copolymerized for 24 h in chlorobenzene with a 1:1 molar ratio. At the end of the reaction a highly viscous solution was formed, which was considerably more viscous than the homopolymerization of VBTOP-TS under the same reaction conditions. For the homopolymerization of VBTOP-SS under the same reaction conditions, on the other hand, a gel was formed after reaction. This result is consistent with the generation of stronger network formation using bridged ion-pairs. After extraction of the unreacted ionic monomer in hexane and drying under vacuum at high temperature (80 °C), a gravimetric yield of over 94% was observed, which indicates almost complete incorporation of the ionic monomer into the polymer. These polyelectrolyte polymers were systematically named as PIPC-CM-*y*-*z*, where *y* and *z* are the mol fraction of VBTOP-SS and VBTOP-TS, respectively. Table 1 summarizes the synthesis information of these polymers.

**Figure 2:**
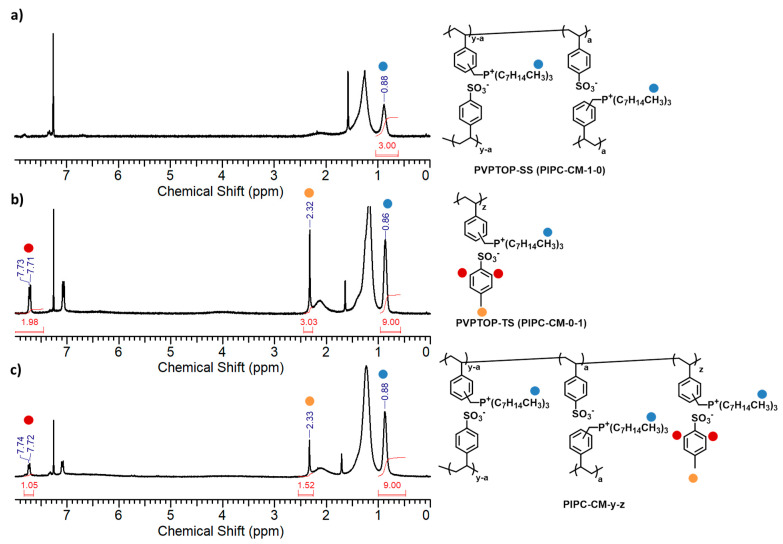
^1^H-NMR (nuclear magnetic resonance) (300 MHz) spectrum of polyelectrolyte copolymers in CDCl_3_: (**a**) PIPC-CM-1-0; (**b**) PIPC-CM-0-1; (**c**) PIPC-CM-0.5-0.5.

Figure 2 shows the ^1^H-NMR (nuclear magnetic resonance) (300 MHz) spectra of representative samples. For the polymer with 100% IPC units (PIPC-CM-1-0), it was hard to dissolve in deuterated chloroform due to a strong electrostatic interaction across the ion-pairs leading to network formation. No peaks from unreacted ionic monomer were observed, which is consistent with the high gravimetric yield of the polymerization. There are some peaks which show up at lower chemical shift region, and these correspond to the protons from soluble alkyl chains. For the polymer with 100% CM units (PIPC-CM-0-1), it easily dissolved in deuterated chloroform solvent consistent with the weaker electrostatic interactions between the pendant ion-pairs. Due to the good solubility, the ^1^H-NMR (300 MHz) spectrum of PIPC-CM-0-1 displays all the characteristic peaks of the polymer structure as shown in Figure 2b. Figure 2c shows the ^1^H-NMR (300 MHz) spectrum of PIPC-CM-0.5-0.5. Considering the peak at 0.88 ppm corresponds to the three protons in –CH3 of tri-*n*-octyl alky chains, and the peak at 2.33 was assigned to the three protons in –CH3 of pendant *p*-toluenesulfonate group, the mole fraction of VBTOP-SS and VBTOP-TS was calculated as 0.507 to 0.493, which was almost identical to the feeding ratio. The mole fraction of IPC to CM units in other polymers (PIPC-CM-0.75-0.25 and PIPC-CM-0.25-0.75) are consistent with this result.

Scheme 1b depicts the synthetic route for the preparation of poly(*n*-butyl acrylate) ionomers through the copolymerization of the ionic monomers VBTOP-SS and/or VBTOP-TS with *n*-butyl acrylate (n-BA) by RAFT polymerization. As an example, n-BA, VBTOP-SS, and VBTOP-TS were copolymerized for 24 h in chlorobenzene with a 100:5:5 molar ratio, respectively. At the end of the reaction a highly viscous solution was formed, which was considerably more viscous than the homopolymerization of n-BA at the same n-BA:RAFT agent ratio. For the copolymerization 100:10 n-BA:VBTOP-SS at the same n-BA-RAFT agent ratio a gel was formed after reaction. This result is consistent with the generation of a stronger network using direct ion-pairs to bridge chains. After extraction of the unreacted ionic monomer in hexane and drying under vacuum at high temperature (80 °C), it was observed that all the ionic polymers have a gravimetric yield over 95%, which indicates the almost complete polymerization of the monomers. These ionic polymers were named as PBA-IPC-CM-*x*-*y*-*z*, where *x*, *y*, and *z* are the mol fraction of the BA, IPC, and CM in the monomer feed, respectively. Our previous paper has showed the ^1^H-NMR (300 MHz) spectrum of PBA-IPC-CM-10-1-0 has 10.1 mol% IPC units to n-BA units, nearly identical to the feed ratio. [17] The mole fraction of IPC and CM in the other PBA-IPC-CM-*x-y-z* samples also obeys this result. Table 2 summarizes the synthesis information and molecular information of these synthesized PBA-IPC-CM copolymers.

### 2.2. Thermo-Mechanical Behavior

**Figure 3:**
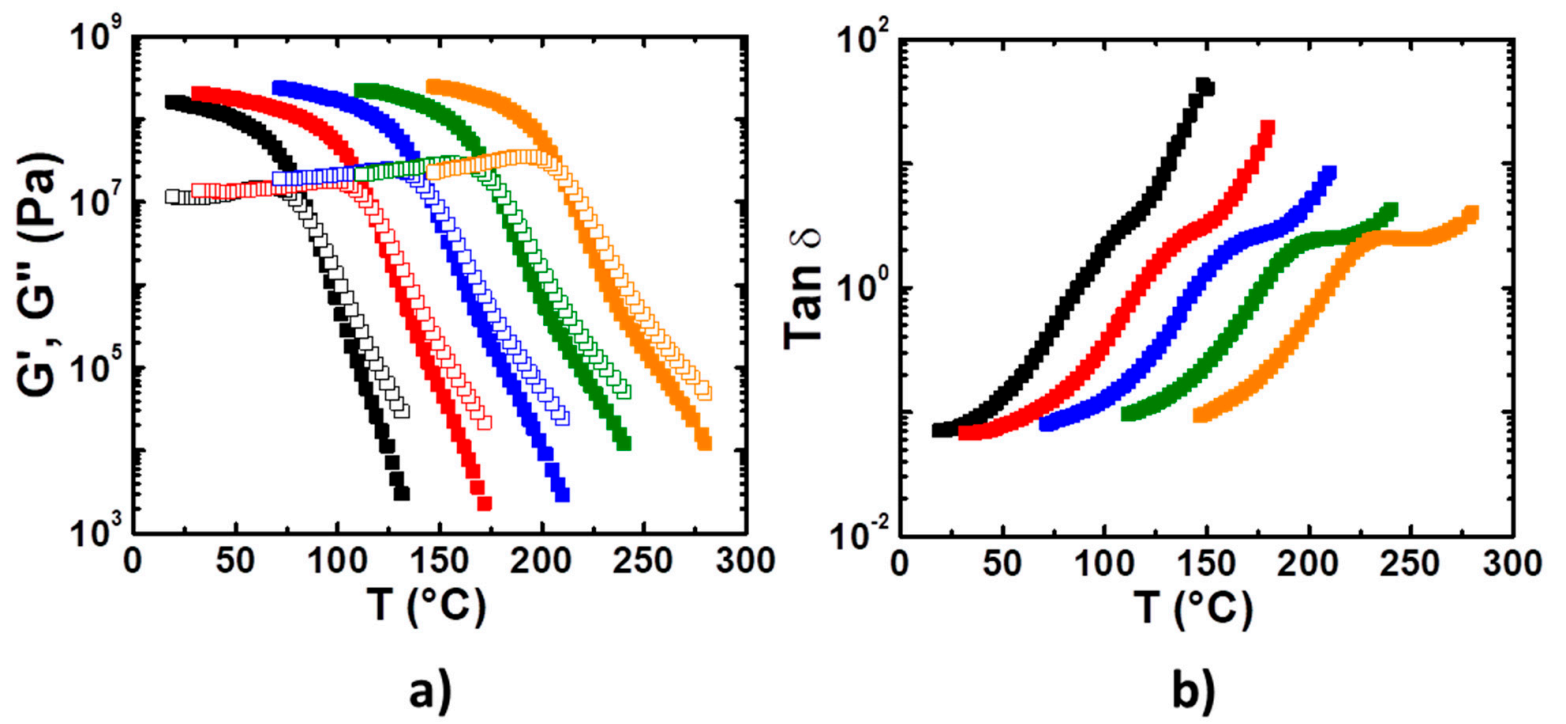
Isochronal temperature sweep of (**a**) *G*′ (filled symbols) and *G*″ (empty symbols); (**b**) loss tangent (*tan δ*) for polyelectrolytes with different ion pair comonomer (IPC) composition: PIPC-CM-1-0 (orange curves); PIPC-CM-0.75-0.25 (green curves); PIPC-CM-0.5-0.5 (blue curves); PIPC-CM-0.25-0.75 (red curves); PIPC-CM-0-1 (black curves). All data were measured at 1 Hz at strains in the linear viscoelastic region. PIPC-CM-1-0 and PIPC-CM-0-1 previously reported in reference [33].

Figure 3 shows the isochronal temperature sweeps of the dynamic moduli and loss tangent for the IPC-CM series with varying IPC concentration. For PIPC-CM-1-0 with 100% IPC units, the *G*’ is much higher than *G*” in the lower temperature region, indicating a solid-like behavior. The high value of *G*’ over 10^8^ Pa suggests the solid behavior is due to the vitrification of the sample to a glass. Upon increasing temperature, a transition was observed from a glassy solid to a viscoelastic liquid with a rapid decrease of moduli and a crossover to *G*” larger than *G*’. A maximum of *G*” was observed at 191 °C during the transition corresponding to the glass transition of PIPC-CM-1-0, which is consistent with the value measured from DSC (189 °C), as previously reported [33]. In the loss tangent (*tan δ*) vs. temperature, a maximum peak was also observed at 236 °C. The shapes of the other curves are self-similar to PIPC-CM-1-0 in the low temperature region with a plateau in *G’* at lower temperature region, in which the magnitude of the plateau slightly increases with increasing IPC concentration. Upon increasing temperature past the glass transition temperature the polymers showed some variation in the dynamic moduli vs. temperature. For PIPC-CM-0-1 with 0% IPC, the temperature range of the shoulder in the loss tangent vs. temperature is very short, which means that the sample transitions almost directly to a terminal fluid region after the glass transition. In other words, there is only one dominant segmental relaxation mode in the PIPC-CM-0-1 sample with 0% IPC content, which indicates there is almost no second relaxation mode from electrostatic interaction or entanglement effects at this molecular weight. Increasing the IPC content leads to a plateau in the loss tangent, which indicates the appearance of second relaxation mode due to the introduction of IPC units in the systems. This rheological complexity in this region has been reported to be observed in the ionomer systems due to the overlapping of chain relaxation in passing through *T_g_* and electrostatic relaxation. [33,43] In the PIPC-CM-0-1 sample, above the glass transition that the electrostatic interactions from dipole-dipole interactions between ion-pairs are weak and have little influence on the overall relaxation. On the other hand, in PIPC-CM-1-0 the ion-pair is much stronger that the dipole–dipole interactions and affects the viscoelastic properties above the glass transition and delays terminal relaxation to a higher temperature.

**Figure 4:**
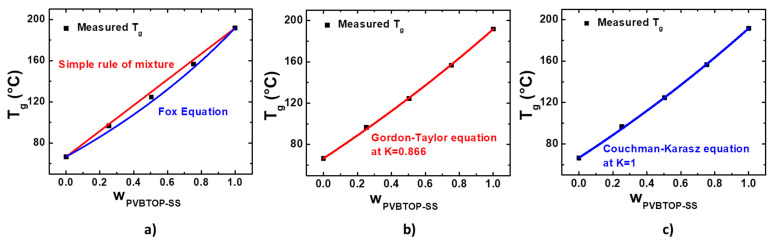
The measured (black solid dot) *T_g_* from rheology and predicted *T_g_* as a function of varying IPC concentration (*w_PVBTOP-SS_*) in polyelectrolytes series based on (**a**). (**a**) Rule of mixtures (red solid line) and Fox equation (blue solid line); (**b**) Gordon–Taylor equation; (**c**) Couchaman–Karasz equation. The *T_g_* was determined as the temperature at the maximum *G*” from temperature sweep.

In addition to variation in the high temperature viscoelastic behavior, the glass transition temperature systematically varies with IPC content. Here, the glass transition temperature of the PIPC-CM’s was defined as the temperature where *G”* is maximum. For PIPC-CM-0-1, it exhibits a *T_g_* of 66 °C, which is lower than the non-ionic polystyrene. The large pendant counter-ions form weaker electrostatic interaction, and the bulky nature of the tri-*n*-octyl phosphonium group act an internal plasticizer to lower *T_g_*. A similar phenomenon was reported by Weiss and coworkers in studying the glass transition temperatures of sulfonated polystyrene ionomers containing varying sizes of alkyl ammonium counterions. [26] In comparison PIPC-CM-1-0 has a glass transition temperature of 191 °C, which is much higher than the non-ionic polystyrene bearing similar chain structure. This result suggests a much stronger electrostatic interaction from bridged ion pairs, which act as temporary crosslinks to restrict the molecular mobility, thus leading to an increased *T_g_*. The other PIPC-CM systems all display one unique *T_g_* suggesting the successful copolymerization of these two ionic monomers and no macro-phase separation after copolymerization. There is a clear trend that the *T_g_s* of the PIPC-CMs increase with IPC concentration (Figure 4). Many approaches have been proposed for estimating the glass transition temperature of amorphous miscible mixture and random copolymers. For a binary mixture, an approximate relationship between the *T_g_* of a miscible mixture and composition is given by the simple rule of mixtures. For amorphous random copolymer systems, the Fox equation has been widely applied for predicting the glass transition of components with very weak or no intermolecular interaction. [44] For comparison the *T_g_* of the ionic copolymers vs. the weight fraction of VTOP-SS segments in the copolymer (*w_VBTOP-SS_*) was calculated using the *T_g_s* of the PVBTOP-SS (PIPC-CM-1-0) and PVBTOP-TS (PIPC-CM-0-1) homopolymers by the simple rule of mixtures,

(1) $$T_{g}=\left(1-w_{VBTOP-SS}\right)T_{g,VBTOP-TS}+w_{VTOP-SS}T_{g,VBTOP-SS}$$

where *T_g,PVBTOP-TS_* and *T_g,PVBTOP-SS_* are the *T_g_s* of the pure PVBTOP-SS and PVTOP-SS, respectively, and the Fox equation,

(2) $$\frac{1}{T_{g}}=\frac{1-w_{VBTOP-SS}}{T_{g,VBTOP-TS}}+\frac{w_{VBTOP-SS}}{T_{g,VBTOP-SS}}$$

Both calculated curves are plotted as solid lines in Figure 4a. The measured *T_g_s* are in between the two predicted curves. The Fox equation is an empirical relation, which displays its limits in some cases for predicting the *T_g_* of amorphous random copolymers. Introducing a fitting parameter leads to a more generalized relationship for predicting the *T_g_* of amorphous random copolymers, i.e., the Gordon–Taylor equation, [45,46]

(3) $$T_{g}=\frac{w_{VBTOP-SS}T_{g,VBTOP-SS}+Kw_{VBTOP-TS}T_{g,\;VBTOP-TS}}{w_{VBTOP-SS}+Kw_{VBTOP-TS}}$$

where the parameter *K* is and adjustable fitting parameter also related to the physical properties as

(4) $$K=\frac{\rho_{VBTOP-SS}}{\rho_{VBTOP-TS}}\frac{∆\alpha_{VBTOP-TS}}{∆\alpha_{VBTOP-SS}}$$

where *ρ_VBTOP-SS_* and *ρ_VBTOP-TS_* are the densities of the VBTOP-SS and VBTOP-TS segments and Δ*α_VBTOP-SS_* and Δ*α_VPTOP-TS_* are the change in the thermal expansion coefficient of the components from glass to rubber (liquid) state. It was found that the Gordon–Taylor equation fits the *T_g_s* of the PIPC-CM series very well with *K* = 0.866 as shown in Figure 4b. In this way, the *T_g_s* of PIPC-CM with varying IPC content could be easily predicted and tuned by different synthesis formulation. An alternative approach for predicting the *T_g_s* of amorphous random copolymer is through the Couchman–Karasz equation, [47]

(5) $$lnT_{g}=\frac{w_{VBTOP-SS}lnT_{g,VBTOP-SS}+Kw_{VBTOP-TS}lnT_{g,VBTOP-TS}}{w_{VBTOP-SS}+Kw_{VBTOP-TS}}$$

where *K* is,

(6) $$K=\frac{∆C_{p,VBTOP-SS}}{∆C_{p,\;VBTOP-TS}}$$

where Δ*C_p,x_* is the change of the heat capacity of component *x* when crossing from the glassy to rubber (liquid state). The Couchman–Karasz equation was found to predict the glass transition temperatures of PIPC-CMs very well as shown in Figure 4c. The parameter *K* was fitted as 1, which indicates identical values of the change of the heat capacity of PVBTOP-SS and PVBTOP-TS homopolymer from glass to rubber (liquid) state. In other words, Δ*C_PVBTOP-SS_* = Δ*C_PVBTOP-TS_* and the Couchman–Karasz equation can be simplified as

(7) $$lnT_{g}=w_{VPTOP-SS}lnT_{g,VBTOP-SS}+w_{VBTOP-SS}lnT_{g,VBTOP-TS}$$

This simplified equation is a commonly used empirical relation named as a logarithmic rule of mixtures, which has been shown to be very successful for fitting the glass transition temperature of many amorphous random copolymers. Therefore, in both methods, the *T_g_s* of polyelectrolyte polymers with varying IPC content could be targeted through the monomer feed ratio. By substituting the IPC with the CM in the monomer feed, less chain bridging ion-pairs are formed, which increases the mobility of the polymer and therefore lowers the *T_g_* compared to the pure polymerized IPC. The trend of *T_g_* vs. IPC content is also consistent with the recent results in polyelectrolyte complexes [2], where *T_g_* increases as the fraction of intrinsic (i.e., chain bridging) ion pairs increases.

**Figure 5:**
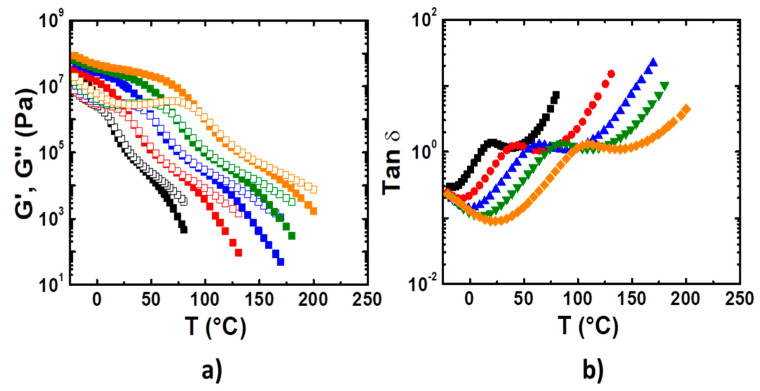
Isochronal temperature sweep of (**a**) *G* (filled symbols) and *G(* (empty symbols); (**b**) loss tangent (tan δ) for poly(*n*-butyl acrylate) ionomer networks (PBA-IPC-CM-*x*-*y*-*z*) with different ion pair comonomer (IPC) composition: PBA-IPC-CM-10-1-0 (orange curves); PBA-IPC-CM-10-0.75-0.25 (green curves); PBA-IPC-CM-10-0.5-0.5 (blue curves); PBA-IPC-CM-10-0.25-0.75 (red curves); PBA-IPC-CM-10-0-1 (black curves). All data were measured at 1 Hz at strains in the linear viscoelastic region. PBA-IPC-CM-10-1-0 and PBA-IPC-CM-10-0-1 previously reported in reference [33].

This plasticizing effect can be also applied to tune the glass transition temperature of ionomers prepared by the copolymerization of the same ionic monomers (VBTOP-SS and VBTOP-TS) with *n-*butyl acrylate. Figure 5 shows the isochronal temperature sweeps of dynamic moduli and loss tangent for the poly (*n*-butyl acrylate) ionomer series with different IPC concentration. For PBA-IPC-CM-10-1-0 *G*’ is much higher than *G*” at the lower temperature region. Our previous research has shown that this is a rubbery plateau due to the physical crosslinking of the PBA-rich matrix by vitrified micro-phased ion-rich domains. Upon increasing temperature, a transition was observed from the solid state to viscoelastic liquid with a rapid decrease of moduli and *G*” larger than *G*’. A maximum of *G”* was observed at 65 °C during the transition corresponding to the glass transition of ion-rich domains, though the *T_g_* could not be observed from DSC measurement. In the loss tangent against temperature, a maximum peak was also observed at 109 °C as shown in Figure 5b. Before entering the terminal region, there is an intermediate region where *G’* ≈ *G*” between the plateau and the onset of terminal relaxation. As we discussed previously, this rheological complexity in the intermediate is related with the segmental chain dynamics of microphase separated ion-rich domains with ion-pair disassociation (ion-hopping), which has been observed in in other ionomer systems where electrostatic interactions contribute to the chain relaxation. The shapes of the other curves are qualitatively similar to the PBA-IPC-CM-10-1-0 and compress horizontally as the IPC concentration decreases. For PBA-IPC-CM-10-0-1 *G*’ is slightly higher than *G*” at low temperature region, which indicates the formation of weak solid network. As only the cations are bound to the polymer backbone in PBA-IPC-CM-10-0-1, the electrostatic interaction is relatively weaker compared to the direct ion-pair crosslinks; thus, the vitrified ion-rich domains would relax much faster upon increasing temperature. This explains why *G’* decreases rapidly with temperature in the plateau region for this sample. Additionally, since the *T_g_* of ion-rich domains in PBA-IPC-CM-10-0-1 should be much lower than the *T_g_* of pure IPC-CM-0-1 due to plasticization by PBA units, this could lead to the overlapping the segmental relaxations of pure PBA domains and ion-rich domains. Therefore, there was no maximum *G”* that could be observed because the solid network is relatively weak. From isochronal temperature sweep of loss tangent; however, a characteristic maximum peak at 20.5 °C indicates a *T_g_* of the ion-rich domains at sub-ambient temperature. There is clear trend that the *T_g_s* increase with IPC concentration, which indicates the plasticization effect of CM unit in the polyacrylate ionomer networks.

**Figure 6:**
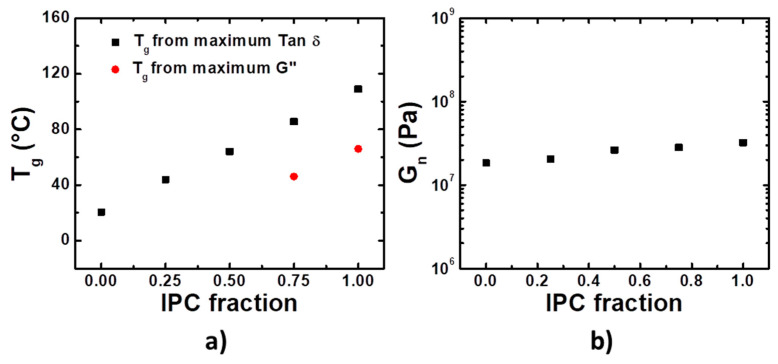
(**a**) The glass transition temperature (*T_g_*) of the ion-rich domains in PBA-IPC-CM series as a function of IPC fraction; (**b**) the plateau modulus (*G_n_*) of PBA-IPC-CM series as a function of IPC fraction. The *T_g_* was determined as the corresponding temperature at the maximum loss tangent (black squares) or the maximum in *G*” (red circles). *G_n_* was determined as the corresponding *G*’ at the minimum loss tangent in the rubbery region.

As discussed previously, the ion-rich domains are a mixture of ionic units and PBA units, and it is hard to measure the exact weight fraction of them, thus the prediction of *T_g_s* of the ion-rich domains remain relatively difficult. Keeping the overall ionic concentration fixed at a 1:10 ratio of ionic:n-BA units, the *T_g_s* of ion-rich domains in PBA-IPC-CM series are found to linearly increase with IPC concentration as shown in Figure 6a. Here both the temperature at the maximum in the loss tangent and in *G”* were plotted as a peak in *G”* could not be observed at lower IPC fractions. Therefore, the glass transition temperatures of vitrified ion-rich domains in the PBA ionomers may also be systematically tuned. At the same time, the plateau modulus (*G_n_*) of these PBA-IPC-CM series remains similar, and slightly increases with IPC concentration due to the stronger electrostatic interactions with IPC concentration (Figure 6b). In that way, the viscoelastic properties can be fine-tuned by different formulation for different applications (e.g., adhesives, elastomers, and shape-memory polymers).

## 3. Conclusions

In this study, the organic ion-pair comonomer (IPC) (VBTOP-SS) and a chemically similar cationic monomer (CM) (VBTOP-TS) were synthesized through quaternization and ion-exchange reaction. These ionic monomers were further copolymerized to prepare polyelectrolytes with varying feeding ratios. The structure-property relationships of this series of polymers was investigated as function of IPC concentration and the CM was found to effectively internally plasticize the polyelectrolyte relative to the pure IPC homopolymer. The Couchman–Karasz equation and Gordon–Taylor equation were found to fit the *T_g_* very well in these series of polymers. This plasticizing effect through the incorporation of pendant ion-pairs could also be applied to the polyampholyte ionomer systems through the copolymerization of *n*-butyl acrylate with the IPC and CM (VBTOP-SS and VBTOP-TS). In this way, the physical properties (e.g., *T_g_*, rubbery plateau modulus, etc.) were tuned at varying IPC composition. Additionally, the *T_g_* of vitrified ion-rich domains in polyampholyte ionomer systems can be approximately predicted by a linear relation with IPC concentration, which opens up the toolbox for designing stimuli-responsive polymers with variable transition temperatures.

## 4. Materials and Methods

Materials and Polymer Synthesis: Hexane (98.5%, Sigma-Aldrich, St. Louis, MO, USA) and chlorobenzene (Cl-Bz, >99%, Sigma-Aldrich, St. Louis, MO, USA) were used as received. *n*-Butyl acrylate (*n*-BA, >98.0%, stabilized, Sigma-Aldrich, St. Louis, MO, USA) was purified by filtering through a column of basic alumina. 2,2′-Azobis(2-methylpropionitrile) (AIBN, Sigma-Aldrich, St. Louis, MO, USA) was purified by dissolving in methanol at 45 °C and recrystallization in a freezer. The nonionic RAFT agent, benzyl dodecyl trithiocarbonate (BDTC), was synthesized using a previously reported method. [48] The ionic monomers vinylbenzyl trioctyl phosphonium 4-styrenesulfonate (VBTOP-SS) and vinylbenzyl tri-*n*-octylphosphonium *p-*toluene sulfonate (VBTOP-TS) were prepared by a two-step synthetic route as previously reported [33].

Polyelectrolyte copolymers were prepared by the copolymerization of the ion-pair comomoner VBTOP-SS and the cationic monomer VBTOP-TS. As an example of a typical reaction, a mixture of the VBTOP-SS (0.336 g, 0.5 mmoL), VBTOP-TS (0.330 g, 0.5 mmoL), BDTC (9.2 mg, 0.025 mmoL) and AIBN (0.82 mg, 0.05 mmoL) was dissolved in chlorobenzene (2.98 g, 2.7 mL) in a 20 mL septum-capped reaction vial with a stir bar. The mixture was sparged with nitrogen for 15 min, and subsequently stirred at 65 °C for 24 h in a thermostatic aluminum reaction block. After polymerization, the reaction vial was quenched to room temperature by running under tap water. The crude product was concentrated under vacuum and immersed in hexane overnight to diffuse out any unreacted monomer. The sedimented product was recovered after decanting the clear liquid. The obtained product was further dried under vacuum at 80 °C for two days to obtain the final product. Yield: 95.3%.

Poly(*n*-butyl acrylate) ionomers were prepared through the copolymerization of ionic monomers (VBTOP-SS & VBTOP-TS) with n-butyl acrylate (n-BA) using reversible addition-fragmentation chain-transfer (RAFT) polymerization. As a typical example, a mixture of the VBTOP-SS (0.336 g, 0.5 mmoL), VBTOP-TS (0.330 g, 0.5 mmoL), *n*-butyl acrylate (1.28 g, 10.0 mmoL), BDTC (18.4 mg, 0.05 mmoL), and AIBN (1.64 mg, 0.01 mmoL) was dissolved in chlorobenzene (2.98 g, 2.7 mL) in a 20 mL septum-capped reaction vial with a stir bar. The mixture was sparged with nitrogen for 15 min, and subsequently stirred at 65 °C for 24 h in a thermostatic aluminum reaction block. After polymerization, the reaction vial was quenched to room temperature by running under tap water. The crude product was concentrated under vacuum and immersed in hexane overnight to diffuse out any unreacted monomer. The sedimented product was recovered after decanting the clear liquid. The obtained product was further dried under vacuum for two days at 80 °C to obtain the final product. Yield: 95.1%.

NMR Characterization: ^1^H nuclear magnetic resonance (^1^H-NMR) (Varian Mercury-300 MHz spectrometer, Varian, Palo Alto, CA, USA) was used to characterize the synthesized materials using deuterated chloroform (CDCl_3_) or deuterated acetone ((CD_3_)_2_CO) as the solvent with a concentration of 10−15 mg/mL for each analyte.

Rheology Measurements: Rheological measurements were performed on a strain-controlled Advance Rheometric Expansion System (ARES), model G2 (TA Instruments, New Castle, DE, USA), in which a parallel plate fixture with a diameter of 8 mm and a gap ~1.0 mm was used. Prior to each experiment, solid samples were prepared by compression molding at elevated temperature (150–280 °C) under vacuum for around 1 h, while the fluid samples were directly loaded at 100 °C and held for 10 min in the fixture before. For the temperature sweep, the samples were ramped from elevated temperature to −20 °C under nitrogen with a constant cooling rate of 5 °C/min. The upper limit temperature of the experiment was set to 300 °C to ensure no degradation of the samples.

## Figures and Tables

**Table 1:** Formulation parameters and polymerization results of PIPC-cationic monomer (CM) polyelectrolyte copolymers.

Sample	VBTOP-SS (mmoL)	VBTOP-TS (mmoL)	RAFT (mmoL)	AIBN (mg)	BzCl (g)	State after Reaction	Yield
PIPC-CM-1-0	1.0	0	0.025	0.82	2.98	Gel	98%
PIPC-CM-0.75-0.25	0.75	0.25	0.025	0.82	2.98	Gel	94%
PIPC-CM-0.5-0.5	0.5	0.5	0.025	0.82	2.98	Solution	95%
PIPC-CM-0.25-0.75	0.25	0.75	0.025	0.82	2.98	Solution	98%
PIPC-CM-0-1	0	1.0	0.025	0.82	2.98	Solution	94%

**Table 2:** Formulation parameters and polymerization results of PBA-IPC-CM copolymers.

Sample	VBTOP-SS (mmoL)	VBTOP-TS (mmoL)	n-BA (mmoL)	RAFT (mmoL)	AIBN (mg)	BzCl (g)	State after Reaction	Yield
PBA-IPC-CM-10-1-0	1.0	0	10	0.05	1.64	2.98	Gel	98%
PBA-IPC-CM-10-0.75-0.25	0.75	0.25	10	0.05	1.64	2.98	Gel	96%
PBA-IPC-CM-10-0.5-0.5	0.5	0.5	10	0.05	1.64	2.98	Solution	97%
PBA-IPC-CM-10-0.25-0.75	0.25	0.75	10	0.05	1.64	2.98	Solution	96%
PBA-IPC-CM-10-0-1	0	1.0	10	0.05	1.64	2.98	Solution	95%

## Data Availability

Data available on request from the corresponding author.
